# Overcoming hypoxia-induced tumor radioresistance in non-small cell lung cancer by targeting DNA-dependent protein kinase in combination with carbon ion irradiation

**DOI:** 10.1186/s13014-017-0939-0

**Published:** 2017-12-29

**Authors:** Carmen Klein, Ivana Dokic, Andrea Mairani, Stewart Mein, Stephan Brons, Peter Häring, Thomas Haberer, Oliver Jäkel, Astrid Zimmermann, Frank Zenke, Andree Blaukat, Jürgen Debus, Amir Abdollahi

**Affiliations:** 1 Division of Molecular and Translational Radiation Oncology, National Center for Tumor Diseases (NCT), Heidelberg University Hospital, Heidelberg, Germany; 2 German Cancer Consortium (DKTK), Heidelberg, Germany; 3 Heidelberg Institute of Radiation Oncology (HIRO), German Cancer Research Center (DKFZ), Heidelberg, Germany; 4 Heidelberg Ion-Beam Therapy Center (HIT), Heidelberg, Germany; 5 National Center for Oncological Hadrontherapy (CNAO), Pavia, Italy; 6 Medical Physics in Radiation Therapy, German Cancer Research Center (DKFZ), Heidelberg, Germany; 7 Merck, Darmstadt, Germany; 8 Division of Molecular and Translational Radiation Oncology, Heidelberg Ion-Beam Therapy Center (HIT), Im Neuenheimer Feld 450, 69120 Heidelberg, Germany

**Keywords:** Hypoxia, Lung cancer, Radioresistance, DNA-Pk, ATM, Serine/threonine kinase inhibitors, Carbon ions

## Abstract

**Background:**

Hypoxia-induced radioresistance constitutes a major obstacle for a curative treatment of cancer. The aim of this study was to investigate effects of photon and carbon ion irradiation in combination with inhibitors of DNA-Damage Response (DDR) on tumor cell radiosensitivity under hypoxic conditions.

**Methods:**

Human non-small cell lung cancer (NSCLC) models, A549 and H1437, were irradiated with dose series of photon and carbon ions under hypoxia (1% O_2_) vs. normoxic conditions (21% O_2_). Clonogenic survival was studied after dual combinations of radiotherapy with inhibitors of DNA-dependent Protein Kinase (DNAPKi, M3814) and ATM serine/threonine kinase (ATMi).

**Results:**

The OER at 30% survival for photon irradiation of A549 cells was 1.4. The maximal oxygen effect measured as survival ratio was 2.34 at 8 Gy photon irradiation of A549 cells. In contrast, no significant oxygen effect was found after carbon ion irradiation. Accordingly, the relative effect of 6 Gy carbon ions was determined as 3.8 under normoxia and. 4.11 under hypoxia. ATM and DNA-PK inhibitors dose dependently sensitized tumor cells for both radiation qualities. For 100 nM DNAPKi the survival ratio at 4 Gy more than doubled from 1.59 under normoxia to 3.3 under hypoxia revealing a strong radiosensitizing effect under hypoxic conditions. In contrast, this ratio only moderately increased after photon irradiation and ATMi under hypoxia. The most effective treatment was combined carbon ion irradiation and DNA damage repair inhibition.

**Conclusions:**

Carbon ions efficiently eradicate hypoxic tumor cells. Both, ATMi and DNAPKi elicit radiosensitizing effects. DNAPKi preferentially sensitizes hypoxic cells to radiotherapy.

**Electronic supplementary material:**

The online version of this article (10.1186/s13014-017-0939-0) contains supplementary material, which is available to authorized users.

## Background

Tumor hypoxia is a critical factor contributing to acquired radioresistance and therapy failure [1, 2]. In line, tumor hypoxia was shown to correlate with poor prognosis and inferior therapeutic outcome in non-small cell lung cancer (NSCLC) [3, 4]. The lack of oxygen leads to decreased production of reactive oxygen species and consequently to reduced DNA damage after conventional radiotherapy with high energy photons [5, 6]. In vitro studies are usually conducted at relatively high oxygen concentrations (20%) compared to the physiological range of 3–7.4% detected in normal tissues [7]. In tumor tissues, average oxygen levels are found to be even lower than 2%, hence referring to a hypoxic microenvironment [8]. There is a relationship between decreased oxygen tension and gradual decline of radiation cell killing changing with different radiation qualities [9]. However, relevant in vitro data on oxygen enhancement ratio (OER) measurements are sparse [10] but urgently needed to adjust treatment planning with more faithful oxygen concentrations and to evaluate the impact of different radiation qualities and multimodal treatments.

Induction of DNA damage is a hallmark of radiation induced cell toxicity [11]. The relevant, lethal DNA damage, complex double-strand breaks (DSB), are repaired by two major pathways, i.e., homologous recombination and non-homologous end-joining. For each of these pathways, members of the phosphotidylinositol-3-kinase (PIK) family are recruited to DSBs: Ataxia-telangiectasia mutated (ATM) and DNA-dependent protein kinase with its catalytic subunit (DNA-PKc). Inhibitors of these key players in DNA damage response (DDR) pathways were shown to enhance the efficacy of radiotherapy [12]. In contrast to sparsely ionizing photon radiation, carbon ions are densely ionizing along their traversal and are considered high linear energy transfer (LET) irradiation [13]. The application of high-LET beams has been shown to increase tumor cell killing by inducing more complex DNA damage that results in an increased radiobiological effectiveness (RBE) [14]. It has been further reported that cell killing by high-LET radiation might be less dependent on the tumor oxygen status [9, 15].

In this study, the effect of carbon ions vs. conventional photon irradiation was investigated on two NSCLC cell lines. We report on the radiosensitizing effect of two novel DDR inhibitors, DNAPKi and ATMi, with photon- and carbon ion irradiation under hypoxic conditions.

## Methods

### Cell lines

The US National Cancer Institute (NCI) offers a panel of 60 human tumor cell lines (NCI-60 panel, https://dtp.cancer.gov/discovery_development/nci-60/) for in vitro evaluations including anti-cancer compounds [16]. From this panel, the NSCLC cell lines A549 and NCI-H1437 (H1437) cells were purchased from the American Type Culture Collection (ATCC). Cells were grown in RPMI 1640 Medium (Gibco) supplemented with 10% Fetal Bovine Serum (FBS) at 37 °C and 5% CO_2_ atmosphere. Experiments in hypoxic conditions were performed at 1% O_2_ and 5% CO_2_.

### Experimental design

**Fig. 1:**
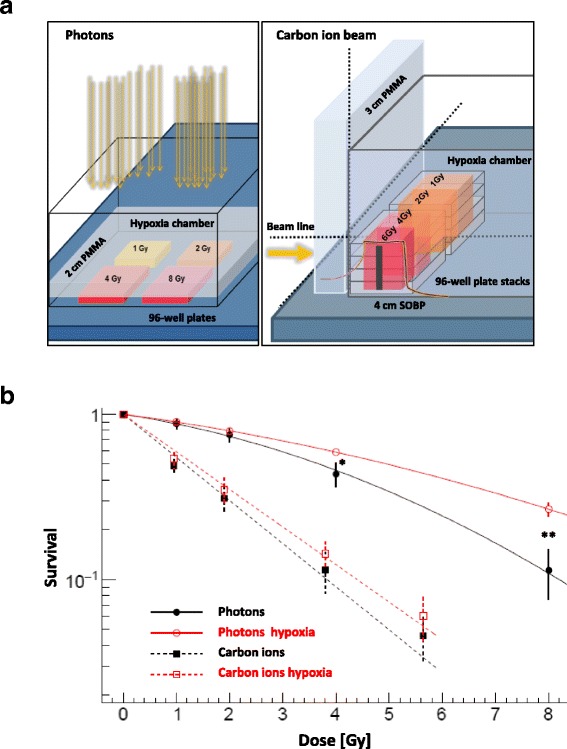
Oxygen effect after photon and carbon ion irradiation. **a** Schematic of the devised hypoxia chamber and 96-well cell culture plates setup for high-throughput clonogenic survival analysis in vertical photon and horizontal carbon ion beam direction. Hypoxia was continuously monitored by sensors for O_2_ and CO_2_ concentrations. **b** Clonogenic survival of the NSCLC cell line A549 irradiated under normoxia (black) and hypoxia (1% O_2,_ red) with photons (solid line) and carbon ions (dashed line). In contrast to carbon irradiation, a significant ratio of survival fractions hypoxia vs. normoxia was found at doses ≥4 Gy after photon irradiation. Consequently, the RBE of carbon ions was enhanced under hypoxic conditions. Bars represent mean ± SD of three independent experiments with n:4 technical replicates each. SOBP: Spread-Out Bragg Peak, PMMA: Poly(methyl methacrylate) for tissue/water-equivalent thickness, arrows: beam direction, *: *p* < 0.05, **: *p* < 0.01

To assess clonogenic survival under hypoxic conditions, we devised a system to irradiate cells in 96-well plates while incubated in a hypoxia chamber (C-chamber; Biospherix) allowing online monitoring of CO_2_ and O_2_ concentrations (ProOx and ProCO2 model; Biospherix) during the entire experiment (Fig. 1a). 50, 100 or 200 A549 cells/well and 100, 200 or 300 H1437 cells /well were seeded up to 16 h before irradiation. Cell numbers increased with escalation of dose and drug concentration. Inhibitors were added to the cells at 50 nM, 100 nM, 200 nM, 500 nM, or 1000 nM for normoxia and at 100 nM, 200 nM, or 500 nM for hypoxia and at 100 nM for carbon ion irradiation. Both ATM and DNA-PK inhibitors were dissolved in DMSO and diluted in RPMI 1640 medium. Controls also contained <0.1% DMSO. To determine the effect of drug treatment alone on clonogenic survival (Plating efficiency, PE), dose series of both compounds at 0, 50, 100, 200, 500, and 1000 nM were analyzed without irradiation. After exposure to inhibitors, cells were incubated for 4 h in hypoxia or in normoxia, respectively. For photon radiation, a vertical beam direction was used to irradiate plates at four different doses. For horizontal carbon ion beams, an irradiation plan was developed to deliver four different doses in SOBP region of the beam.

Cells were irradiated in the hypoxia chamber with a dose series of photons (1, 2, 4, or 8 Gy) and carbon ions (approx. 1, 2, 4, or 6 Gy) and thereafter incubated under normoxic conditions. Inhibitors were left in the media for 24 h and then replaced with fresh RPMI 1640 medium and the plates were returned to the incubator for colony formation. After 4 (A549) or 7 days (H1437) plates were imaged by microscopy at 4× magnification (IncuCyte, Essen Bioscience). The images were analyzed by the IncuCyte Zoom Software (Essen Bioscience) and colony counts were confirmed by manual curation. Counts were normalized to non-irradiated samples at each corresponding baseline inhibitor concentration.

### Dose planning and simulations

Photon planning was done with Raystation treatment planning system (RaySearch Laboratories) based on a CT scan of the hypoxia chamber containing 96-well plates filled with water. Irradiation was performed on a Siemens Artiste (6 MV). For carbon ion delivery, Spread-out Bragg peak (SOBP) was physically optimized with the Treatment Planning for Particles (TRiP98) system, using a 30x30x30 cm^3^ water phantom positioned at 100 cm source-surface distance (SSD) [17]. To reduce physical uncertainties on cell survival introduced by the heterogeneous target, a detailed geometry of the utilized 96-well plates was incorporated into a FLUKA *Monte Carlo* simulation of the Heidelberg Ion Beam Therapy (HIT) beam-line [18]. Dose maps were generated, with dose uniformity found to be within 2% range in the SOBP region. Carbon dose levels for planned 1, 2, 4 and 6 Gy were corrected accordingly to actual prescribed 0.95, 1.9, 3.8, and 5.64 Gy.

### Software and calculations

The survival fractions derived from the clonogenic survival data were fitted according to the linear-quadratic model for photons. A linear model was applied to carbon ion data. The fits as well as OER, RBE, and SER values (Additional file 1: Table S5 and Table S6) were calculated using an in-house tool based on Minuit package available in ROOT [19]. PE values were plotted with GraphPad Prism 5. To display the oxygen effect, the relative effect of carbon ions, and the sensitization effect of inhibitors, measured data points were used to determine ratios of clonogenic survival at a corresponding dose: Ratios were calculated as survival fractions of hypoxic cells and normoxic cells; survival fractions of cells irradiated with photons and cells irradiated with carbon ions; survival fractions of mock-treated cells and cells treated with inhibitors at the same dose, respectively. Effects were compared at a preferential dose of 4 Gy being a reasonable dose for patients in fractionated therapy.

### Statistics

Data are presented as means and standard deviations (SD). Statistical significance was determined using unpaired *t-test* (two-tailed). The asterisks represent significantly different values. Data represent average values of at least three independent experiments, each performed with technical quadruplicates (n:4).

## Results

### Oxygen effect and relative effect for photon vs. carbon irradiation under hypoxia

Hypoxia increased the survival fraction of A549 cells significantly (between 1.36 to 2.34-fold) at photon doses ≥4 Gy under hypoxia vs. normoxia (*p* < 0.05). In contrast, no significant oxygen effect was found after carbon ion irradiation (Fig. 1b, Table 1). The calculated OER at 30% survival was 1.4 for photons and 1.2 for carbon ions (Additional file 1: Table S5A). Accordingly, the survival ratios displaying the relative effect of carbon ion vs. photon irradiation at 4 Gy increased from 3.8 under normoxia to 4.11 at 1% O_2_ concentration (Table 2). Corresponding RBE values calculated from fits at 30% survival are 2.7 under normoxia and 3.1 under hypoxia. The survival data for H1437 cells are presented in the supplemental material.

**Table 1:** Ratio of survival fractions normoxia vs. hypoxia for A549 cells at indicated doses

	Normoxia	Hypoxia	*p*-value	Ratio
SF_4Gy_ Photons	0.44 ± 0.07	0.59 ± 0.02	0.025	1.36 ± 0.18
SF_8Gy_ Photons	0.11 ± 0.04	0.27 ± 0.03	0.004	2.34 ± 0.56
SF_3.8Gy_ Carbon ions	0.11 ± 0.03	0.14 ± 0.03	0.29	1.25 ± 0.13
SF_5.6Gy_ Carbon ions	0.05 ± 0.01	0.06 ± 0.02	0.35	1.32 ± 0.02

*Abbreviations:* SF survival fraction at indicated dose

**Table 2:** Relative effect of photons vs. carbon ions for A549 cells at the indicated dose

	SF_4Gy_			
	Photons	Carbon ions	*p*-value	Ratio
Normoxia	0.44 ± 0.07	0.12 ± 0.03	0.002	3.8 ± 0.20
Hypoxia	0.59 ± 0.02	0.14 ± 0.03	<0.0001	4.11 ± 0.34

*Abbreviations:* SF_4Gy_ survival fraction at 4 Gy photons and 3.8 Gy carbon ions

### Preferential Radiosensitization of hypoxic cells to DNAPKi

**Fig. 2:**
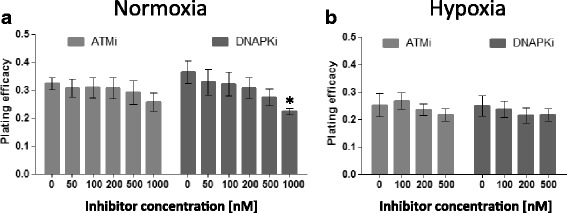
Lack of cytotoxicity of utilized ATMi and DNAPKi alone at pharmacologically relevant doses. PE of A549 cells after treatment with dose series of ATMi (light grey) or DNAPKi (dark grey), respectively, under normoxia (**a**) and hypoxia (**b**). Statistically significant reduction of PE was only found under normoxia for doses ≥1000 nM. Bars represent mean ± SD of three independent experiments with *n*:4 technical replicates each

Next, we investigated the inherent and radiosensitizing effect of two novel DNAPK and ATM serine-threonine kinase inhibitors. The PE was not significantly reduced after ATMi treatment. The PE was only significantly reduced by 15% after 1000 nM of DNAPKi (Fig. 2). This is in line with the reported high selectivity and on target potency of these compounds: DNAPKi (M3814) is a highly potent and selective inhibitor of DNA-PK with subnanomolar potency on its target [20, 21]. The split to closely related PIKK proteins has been measured in biochemical assays and is about 150-fold to PI3K delta and greater than 400-fold to the other family members (ATM, PI3Kalpha – delta, mTOR). The preclinical ATM inhibitor tested is a subnanomolar potent inhibitor with 50-fold selectivity over DNA-PK and greater than 1000-fold selectivity against the other PIKK family members (ATR, PI3Kalpha – delta, mTOR).

**Fig. 3:**
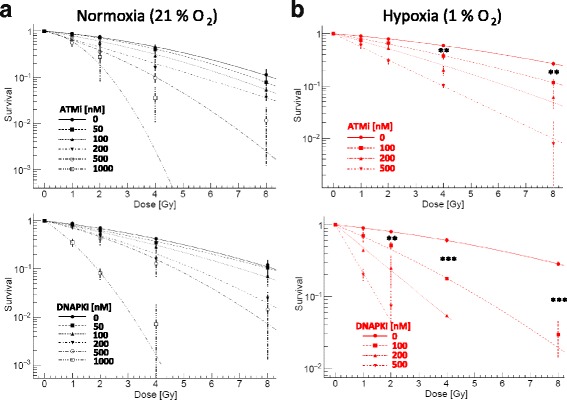
Dose dependent radiosensitizing effect of ATMi and DNAPKi after photon irradiation. Clonogenic survival of A549 tumor cells irradiated with photons under normoxia (**a**) and hypoxia (**b**) in combination with increasing concentrations of ATMi (upper panel) or DNAPKi (lower panel), respectively. Both DDR inhibitors exhibited potent dose dependent radiosensitizing effects. Note, the ratio of survival fractions with and without DNAPKi increased significantly under hypoxic conditions (Table 3). Bars represent mean ± SD of three independent experiments with n:4 technical replicates each. **: p < 0.01; ***: *p* < 0.005

After photon irradiation both compounds exhibited dose dependent radiosensitizing effects under normoxic and hypoxic conditions (Fig. 3). For 100 nM ATMi, the sensitization effect at 4 Gy photons increased from 1.4 under normoxia to 1.6 under hypoxia (Table 3). The radiosensitizing effect of DNAPKi was markedly enhanced under hypoxic conditions. For 100 nM DNAPKi the survival ratio at 4 Gy more than doubled from 1.5 under normoxia to 3.3 under hypoxia (Fig. 3, Table 3). The enhanced sensitizing effect of DNAPKi under hypoxia was found in both cell lines (Additional file 1: Fig. S3).

**Table 3:** Effect of ATMi and DNAPKi for photon irradiation of A549 cells at indicated doses

	Normoxia				Hypoxia			
Photons	0 nM	100 nM ATMi	*p*-value	Ratio	0 nM	100 nM ATMi	*p*- value	Ratio
SF_4Gy_	0.43 ± 0.07	0.29 ± 0.10	0.12	1.48 ± 0.25	0.59 ± 0.02	0.37 ± 0.07	0.007	1.57 ± 0.23
SF_8Gy_	0.11 ± 0.04	0.05 ± 0.01	0.06	2.11 ± 0.31	0.27 ± 0.03	0.12 ± 0.02	0.002	2.30 ± 0.26
		100 nM DNAPKi				100 nM DNAPKi		
SF_4Gy_		0.29 ± 0.07	0.12	1.51 ± 0.09		0.18 ± 0.01	<0.0001	3.34 ± 0.04
SF_8Gy_		0.07 ± 0.03	0.072	1.59 ± 0.14		0.07 ± 0.03	0.0002	9.03 ± 3.83

*Abbreviations:* SF, survival fraction at indicated dose

### Effect of DDR inhibition in combination with carbon ion irradiation

**Fig. 4:**
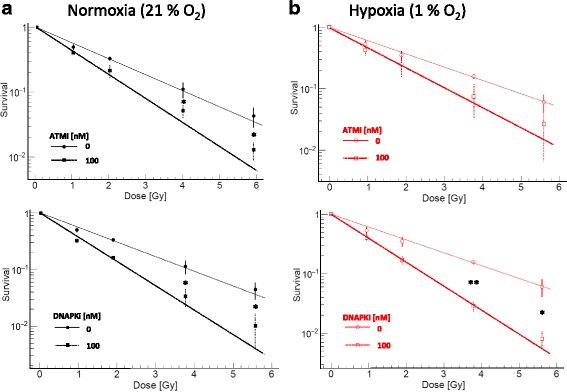
DNAPKi further augments efficient eradication of hypoxic tumor cells by carbon ion. Clonogenic survival data for A549 cells irradiated with carbon ions under normoxia (**a**) and hypoxia (**b**) in combination with 100 nM ATMi (upper panel) or DNAPKi (lower panel), respectively. Both inhibitors radiosensitized carbon ions under normoxia. In line with photon irradiation, with DNAPKi the ratio of survival fractions was further increased after carbon irradiation under hypoxic conditions. Bars represent mean ± SD of three independent experiments with n:4 technical replicates each. *: *p* < 0.05, **: *p* < 0.01

Next, we aimed to discover the potential of DDR inhibitors to further improve eradication of radioresistant hypoxic cells. Based on the PE data demonstrating no relevant cytotoxicity at 100 nM for both inhibitors (Fig. 2), but strong radiosensitizing effects after photon irradiation (Fig. 3), this concentration was selected for combinations with carbon ion irradiation. Both inhibitors exhibit potent radiosensitizing effects in combination with high-LET carbon irradiation (Fig. 4). The SER at 30% survival was 1.5 for 100 nM ATMi and carbon irradiation under normoxic conditions (Additional file 1: Table S5B). This radiosensitizing effect was not further enhanced by addition of ATMi to carbon ions under hypoxic condition. In contrast, the SER at 30% survival increased from 1.7 under normoxia to 1.9 under hypoxia after combined carbon irradiation and DNAPKi. When comparing survival ratios after treatment with DNAPKi and 4 Gy carbon ion irradiation the ratios increased from 3.4 to 5.1 normoxia vs. hypoxia (Table 4). Overall, combination of DNAPKi and carbon irradiation most efficiently eradicated hypoxic NSCLC tumor cells.

**Table 4:** Effect of ATMi and DNAPKi for irradiation of A549 cells with carbon ions at indicated doses

		Normoxia			Hypoxia			
Carbon ions	0 nM	100 nM ATMi	*p*-value	Ratio	0 nM	100 nM ATMi	*p*-value	Ratio
SF_3.8Gy_	0.11 ± 0.03	0.05 ± 0.01	0.03	2.13 ± 0.09	0.14 ± 0.03	0.07 ± 0.04	0.08	1.95 ± 0.64
SF_5.6Gy_	0.05 ± 0.01	0.02 ± 0.002	0.028	2.99 ± 0.59	0.06 ± 0.02	0.03 ± 0.02	0.14	2.15 ± 0.84
		100 nM DNAPKi				100 nM DNAPKi		
SF_3.8Gy_		0.03 ± 0.01	0.013	3.38 ± 0.34		0.03 ± 0.005	0.004	5.07 ± 0.02
SF_5.6Gy_		0.01 ± 0.03	0.021	4.39 ± 1.30		0.008 ± 0.03	0.015	7.07 ± 0.06

*Abbreviations:* SF survival fraction at indicated dose

## Discussion

We report here efficient eradication of hypoxic NSCLC tumor cells, in particular, after combined DNAPKi and ionizing radiation. Both, ATMi and DNAPKi demonstrated a gradual dose dependent radiosensitizing effect under normoxia and hypoxic conditions. Our data further confirmed findings from previous work reporting on a minimal dependency of high-LET carbon beams on cellular oxygen concentration [22, 23]. A combination of DNAPKi and carbon ions most efficiently eradicated hypoxic tumor cells (Fig. 4b).

To investigate irradiation effects under hypoxic conditions, establishing an in vitro experimental approach closest to the in vivo situation was a prerequisite. Current systems for conducting radiation experiments under hypoxic conditions may show some limitations e.g., utilize a strict anoxic environment that may not reflect the moderate hypoxic niche of the radioresistant tumor cell populations [24, 25]. We devised a system that allows to work with standard cell culture plates and conveniently transfer them between normoxia and different online monitored hypoxic conditions such as the 1% O_2_ utilized in this study. Moreover, the high-throughput performance in 96-well format with cell lines that form circumscribed colonies outperforms currently existing petri dish-sized hypoxia systems [26]. With this option, we were able to combine potential radiosensitizers with irradiation in an identical setting under normoxia and hypoxia.

ATM and DNA-PKc are two central constituents of DDR and their inhibition was shown to radiosensitize NSCLC cell lines [27, 28] as well as other tumor entities [29, 30] to photon irradiation under normoxia. However, only little is known about the effect of DNA-PKc inhibition on the radiosensitivity of hypoxic cells. Lindquist and colleagues described radiosensitivity effects with photon irradiation of DNA-PK deficient mouse embryonic fibroblast cells (SCID/st) under normoxic and hypoxic conditions compared to isogenic DNA-PK wildtype cells (CB.17) and that DNA-PK inhibition in combination with photon irradiation decreases the clonogenic survival of both oxic and hypoxic cells [31]. They suggested that impairment of DNA-PK radiosensitizes independently of cellular oxygen status. In contrast, we found a marked enhancement of DNAPKi radiosensitizing effects under hypoxia, in both cell lines, but not for ATM inhibition. One plausible explanation for this phenomenon is that moderate hypoxia may alter DNA repair signaling e.g., by post-translational modifications in a HIF-dependent manner [32]. Our data suggest that the therapeutic window for DNAPKi might be broader than originally anticipated by efficient eradication of the most radioresistant cells residing in the hypoxic tumor niche. This hypothesis, once successfully confirmed by in vivo experiments, could form the basis for rationally designed clinical trials investigating the impact of DNAPKi in otherwise radioresistant hypoxic tumors.

In addition to pharmacological radiosensitization of hypoxic cells, we provide here novel rationale to combine DDR inhibitors with high-LET carbon ion irradiation. The high RBE of carbon ions is attributed to their ability to form more complex unrepairable DSB correlating with large nuclear repair foci and increased number of residual DNA damage [14, 33]. Our results with NSCLC cell lines confirm data for CHO-K1 cells [26] showing that the low dependency of high-LET irradiation on cellular oxygen would further increase the RBE under hypoxic conditions. Additionally, these findings justify the usage of carbon ions to treat patients with hypoxia-associated radioresistant tumors.

We further evaluated the significance of inhibiting DDR with carbon ions. Interestingly, the sensitizing effect of DNAPKi was more pronounced than the effect of ATM inhibition. Moreover, the DNAPKi sensitizing effect was substantially enhanced (between ~50–60%) in survival ratios after carbon irradiation under hypoxic compared to normoxic conditions. On the contrary, the ATMi sensitizing effect remained in the same range under hypoxic vs. normoxic conditions after carbon ion irradiation. It is conceivable that the formation of lethal complex DNA damages is enhanced with higher photon doses, higher LET and inhibition of DNA-PKc. However, further mechanistic studies are needed to dissect the molecular mechanisms underlying the superior radiosensitizing principle behind DNAPKi treatment under hypoxic conditions.

## Conclusion

Dual therapy with DNAPKi and carbon ion irradiation demonstrated the highest efficacy in eradicating otherwise radioresistant hypoxic tumors. Our data suggest potent reversal of the radioresistant phenotype of hypoxic tumors by DNAPKi that warrants further preclinical and clinical evaluation.

## Additional file

Additional file 1:
**S1**-**S4** Clonogenic survival of H1437 cells; In parallel to A549, another NSCLC cell line, H1437, was used in the same setup to compare the oxygen effect as well as sensitizing effects of inhibitors with photon vs. carbon ion irradiations. **S5** Table listing OER, RBE, and SER for A549 and H1437 cells. OER, RBE, and SER were calculated from linear quadratic fits at 30% survival. (PDF 524 kb)

## Abbreviations

ATM: Ataxia-telangiectasia mutated
ATMi: Inhibitor of ATM
DDR: DNA damage response
DNA-PKc: Catalytic subunit of DNA-dependent Protein Kinase
DNAPKi: Inhibitor of DNA-dependent Protein Kinase
HIT: Heidelberger Ionenstrahl-Therapiezentrum
LET: Linear energy transfer
NSCLC: Non-small cell lung cancer
OER: Oxygen enhancement ratio
PE: Plating efficiency
PIK: Phosphotidylinositol-3-kinase
RBE: Relative biological effect
ROS: Reactive oxygen species
SER: Sensitization enhancement ratio
SOBP: Spread-out Bragg peak
SSD: Source-surface distance
