# Supplementary material for: Overcoming hypoxia-induced tumor radioresistance in non-small cell lung cancer by targeting DNA-dependent protein kinase in combination with carbon ion irradiation
Source: Radiat Oncol. 2017 Dec 29;12:208. doi: 10.1186/s13014-017-0939-0 (PMC5747947; doi:10.1186/s13014-017-0939-0)
Supplement: Additional file 1: — S1-S4 Clonogenic survival of H1437 cells; In parallel to A549, another NSCLC cell line, H1437, was used in the same setup to compare the oxygen effect as well as sensitizing effects of inhibitors with photon vs. carbon ion irradiations. S5 Table listing OER, RBE, and SER for A549 and H1437 cells. OER, RBE, and SER were calculated from linear quadratic fits at 30% survival. (PDF 524 kb) [file 13014_2017_939_MOESM1_ESM.pdf]

Supplementary Materials for

Overcoming hypoxia-induced tumor radioresistance in non-small cell lung cancer by targeting DNA-dependent protein kinase in combination with carbon ion irradiation

Carmen Klein[1-4], Ivana Dokic[1-4], Andrea Mairani [1-5], Steward Mein[1-4], Stefan Brons[4], Peter Häring[3], Thomas Haberer[4], Oliver Jäkel[3] Astrid Zimmerman[7], Frank Zenke[7], Andree Blaukat[7], Jürgen Debus[1-4], Amir Abdollahi[1-4*]

[1] Division of Molecular and Translational Radiation Oncology, National Center for Tumor Diseases (NCT), Heidelberg University Hospital, Heidelberg, Germany.

[2] German Cancer Consortium (DKTK), Heidelberg, Germany

[3] Heidelberg Institute of Radiation Oncology (HIRO), German Cancer Research Center (DKFZ), Heidelberg, Germany.

[4] Heidelberg Ion-Beam Therapy Center (HIT), Heidelberg, Germany.

[5] National Center for Oncological Hadrontherapy (CNAO), Pavia, Italy.

[6] Medical Physics in Radiation Therapy, German Cancer Research Center, Heidelberg, Germany.

[7] Merck, Darmstadt, Germany

***Correspondence to:**

Amir Abdollahi, MD, PhD
Division of Molecular and Translational Radiation Oncology
Heidelberg Ion-Beam Therapy Center (HIT)
Im Neuenheimer Feld 450
69120 Heidelberg
Germany

Tel: +49-6221-56-39604
Fax: +49-6221-56-8537
Email: a.amir@dkfz.de; amir.abdollahi@med.uni-heidelberg.de

This PDF file includes:

I. Supplementary Figure S1..page 2

II. Supplementary Figure S2..page 3

III. Supplementary Figure S3..page 4

IV. Supplementary Figure S4..page 5

V. Supplementary Table S5..page 6

VI. Supplementary Table S6..page 7

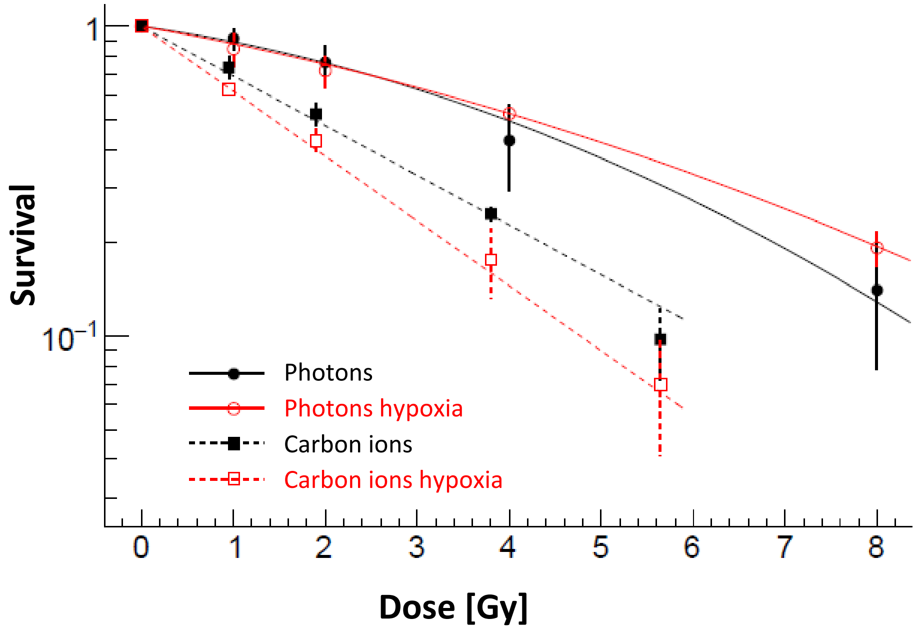

Figure S1. Clonogenic survival of the NSCLC cell line H1437 irradiated under normoxia (black) and hypoxia (1 % $O_{2,}$ red) with photons (solid line) and carbon ions (dashed line). Bars represent mean ± SD of three independent experiments with n=4 technical replicates each.

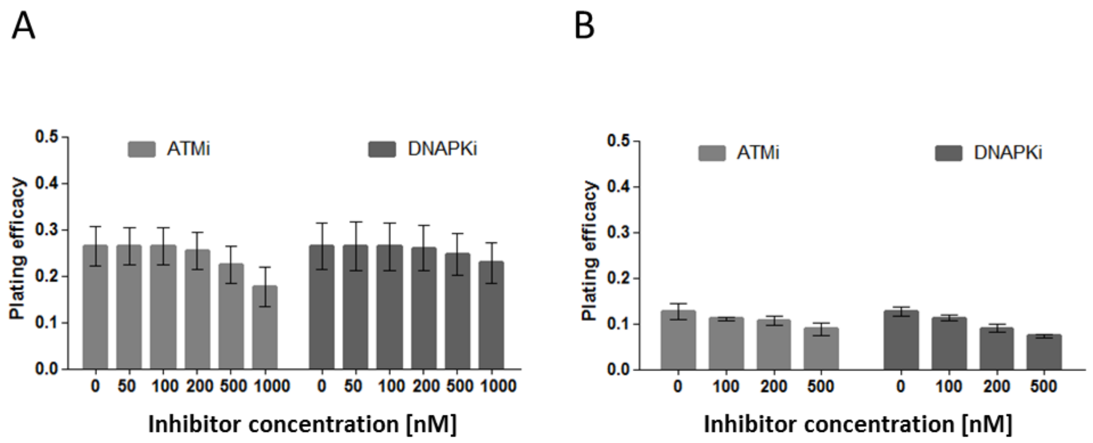

Figure S2. Plating efficacy (PE) of H1437 cells after treatment with dose series of ATMi (grey) or DNAPKi (black), respectively, under normoxia (**A**) and hypoxia (**B**). Bars represent mean ± SD of three independent experiments with n=4 technical replicates each.

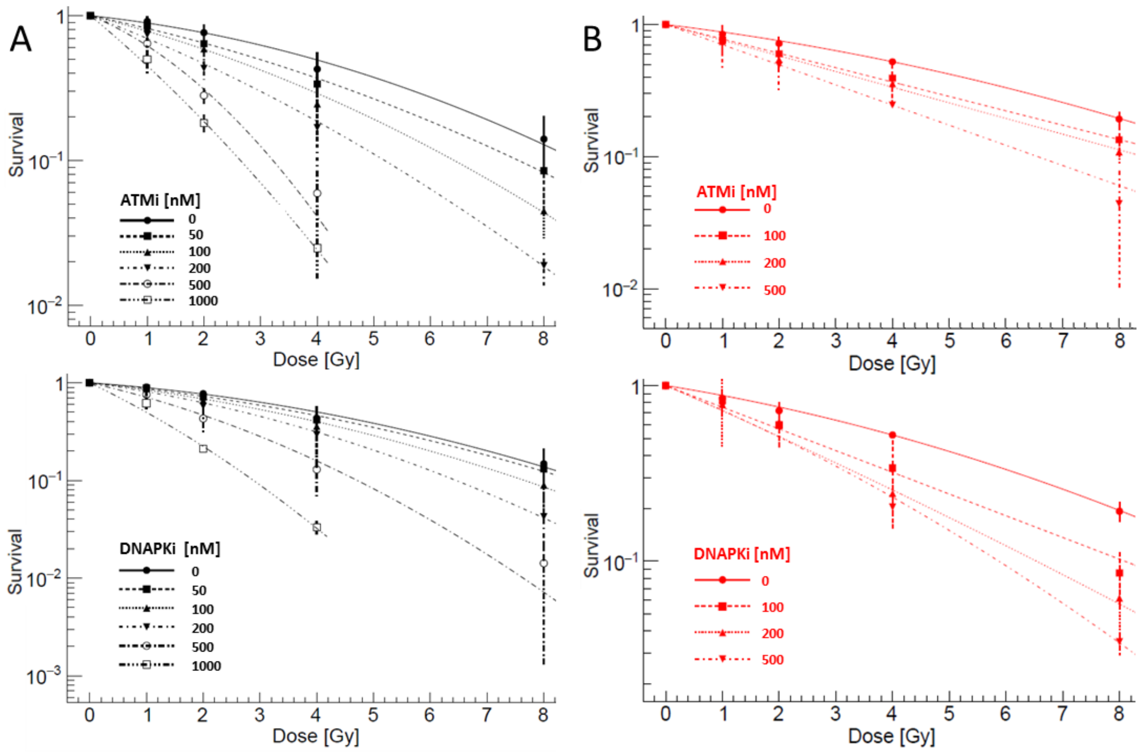

Figure S3. Clonogenic survival of H1437 cells irradiated with photons under normoxia (**A**) and hypoxia (**B**) in combination with increasing concentrations of ATMi (upper panel) or DNAPKi (lower panel), respectively. Data were normalized to non-irradiated samples at each corresponding baseline inhibitor concentration. Bars represent mean ± SD of three independent experiments with n=4 technical replicates each.

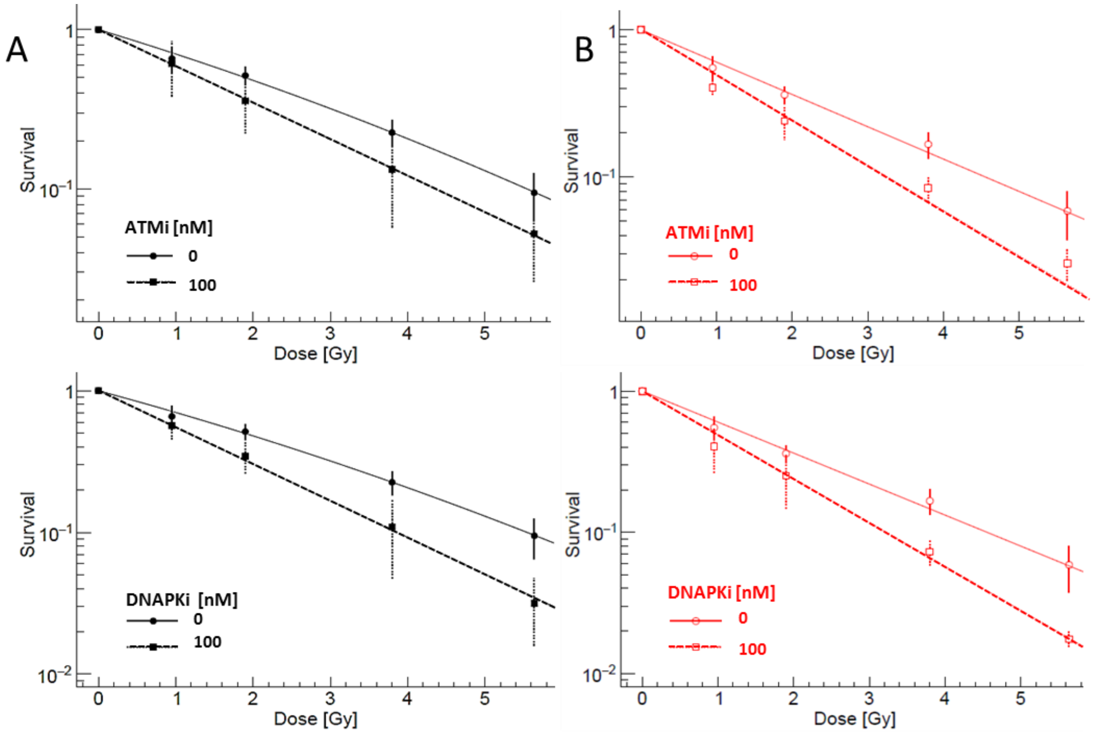

Figure S4. Clonogenic survival data for H1437 cells irradiated with carbon ions under normoxia (**A**) and hypoxia (**B**) in combination with 100 nM ATMi (upper panel) or DNAPKi (lower panel), respectively. Data were normalized to non-irradiated cells treated with 100 nM of the DDR inhibitors (baseline). Bars represent mean ± SD of three independent experiments with n=4 technical replicates each.

Table S5.

A. OER and RBE for A549 cells at 30 % survival fraction.

**OER Photons**	**SEM**	**RBE Normoxia**	**SEM**
**1.39**	**0.22**	**2.65**	**0.35**

**OER Carbon ions**	**SEM**	**RBE Hypoxia**	**SEM**
**1.19**	**0.02**	**3.1**	**0.3**

Abbreviations: OER: oxygen enhancement ratio; RBE: relative biological effectiveness; SEM: Standard error of the mean

B. SER for A549 cells at indicated ATM or DNAPK inhibitor concentrations in combination after photon and carbon ion irradiation under normoxic vs. hypoxic conditions.

**Inhibitor conc. [nM]**	**SER ATMi Normoxia**	**SEM**	**SER ATMi Hypoxia**	**SEM**	**SER DNAPKi Normoxia**	**SEM**	**SER DNAPKi Hypoxia**	**SEM**
**Photons**								
**50**	1.11	0.19			1.20	0.16		
**100**	1.31	0.52	1.64	0.15	1.40	0.19	2.70	0.24
**200**	1.87	0.39	2.16	0.19	1.89	0.25	4.60	0.41
**500**	2.54	0.34	3.63	0.33	2.13	0.28	9.75	0.94
**1000**	3.28	0.49			5.41	0.726		
**Carbon ions**								
**100**	1.50	0.03	1.50	0.06	1.66	0.03	1.86	0.03

Abbreviations: SER: Sensitization enhancement ratio; SEM: Standard error of the mean

Table S6.

A. OER and RBE for H1437 cells at 30 % survival fraction.

OER Photons	SEM	RBE Normoxia	SEM
**1.09**	**0.2136**	**1.92**	**0.2887**

OER Carbon ions	SEM	RBE Hypoxia	SEM
**0.78**	**0.0231**	**2.67**	**0.3522**

Abbreviations: OER: oxygen enhancement ratio; RBE: relative biological effectiveness; SEM: Standard error of the mean

B. SER for H1437 cells at indicated ATM or DNAPK inhibitor concentration in combination with photons and carbon ions under normoxic vs. hypoxic conditions.

Inhibitor conc. [nM]	SER ATMi Normoxia	SEM	SER ATMi Hypoxia	SEM	SER DNAPKi Normoxia	SEM	SER DNAPKi Hypoxia	SEM
**Photons**								
**50**	1.26	0.64			1.06	0.34		
**100**	1.50	0.62	1.32	0.18	1.20	0.42	1.50	0.20
**200**	1.95	1.24	1.44	0.19	1.42	0.47	1.82	0.24
**500**	2.86	0.72	1.85	0.24	2.02	1.36	1.88	2.09
**1000**	3.97	10.83			3.55	2.53		
**Carbon ions**								
**100**	1.34	0.06	1.51	0.06	1.40	0.04	1.41	0.03

Abbreviations: SER: Sensitization enhancement ratio; SEM: Standard error of the mean
